# Board of Health in Cincinnati

**Published:** 1828-07

**Authors:** 


15. *Our Board of Health.*—More than twelve months ago, the City Council of Cincinnati instituted a Board of Health; and delegated to it a variety of advisory and other powers. As yet, we believe, the public have been favoured with no manifestation of its wisdom; but whether from its *vis inertia*, so characteristic of learned bodies, or the previous excellence of our police, rendering all interference superfluous, we are not fully informed. It is believed, however, that the said conservators of the public health, have but seldom been together; and that various parts of the city abound in evidences, that its police regulations are susceptible of improvement. We would respectfully suggest to the Council, the propriety, of constituting the Board, in future, *entirely* of medical gentlemen; who, to avoid the imputation of willingly suffering the public causes of disease to multiply upon society, would be induced to discharge their duties with vigilance, and good will. Meanwhile we are happy to be able to say, that, notwithstanding every appearance of neglect, the city was never healthier, in July, than at present.

